# Variations in Psychiatric Emergency Department Boarding for Medicaid-Enrolled Youths

**DOI:** 10.1001/jamahealthforum.2025.3177

**Published:** 2025-08-15

**Authors:** K. John McConnell, Thomas H. A. Meath, Lindsay N. Overhage

**Affiliations:** 1 Center for Health Systems Effectiveness, Oregon Health & Science University, Portland; 2 Department of Psychiatry, Cambridge Health Alliance, Cambridge, Massachusetts

## Abstract

This cohort study describes US state-level variations in boarding prevalence among Medicaid-enrolled youths with emergency department visits for mental health conditions in 2022.

## Introduction

Emergency department (ED) boarding—the practice of holding patients in the ED while awaiting an inpatient bed—has become an increasing concern for youths with mental health conditions.^1^ Boarding may disproportionately affect youths enrolled in Medicaid, which covers more than 35 million children and adolescents (almost half of all youths in the US).^2^ We investigated the prevalence of and variations in boarding for ED visits for mental health conditions among Medicaid-enrolled youths in 2022.

## Methods

The Oregon Health & Science University Institutional Review Board approved this cohort study. The need for informed consent was waived owing to use of 2022 Medicaid claims data from the Transformed Medicaid Statistical Information System Analytic Files. We excluded states with data quality concerns, resulting in a final sample of 44 US states. The study population included Medicaid enrollees aged 5 to 17 years, excluding those dually eligible for Medicare or with missing or conflicting demographic records. We followed the STROBE reporting guideline.

Mental health–related ED visits were identified by primary diagnosis, using Clinical Classifications Software Refined^3^ codes (MBD001-MBD009, MBD012, MBD027, EXT021, and a modified version of MBD010 excluding developmental feeding diagnoses). Following prior literature, ED boarding was defined as a visit spanning 2 to 6 midnights (length, 3-7 days).^4^ Conditional on any ED visit for these mental health conditions, we calculated the percentage of visits resulting in a boarding event. Analyses were conducted using R, version 4.4.2 (R Project for Statistical Computing).

## Results

Our final sample included 255 139 ED visits for mental health conditions among Medicaid-enrolled youths (35.7% male and 64.3% female; mean [SD] age, 14.6 [2.6] years) in 2022 (Table). Of these, more than 1 in 10 visits (30 359 [11.9%]) resulted in 3 to 7 days of boarding (mean [SD], 4.5 [1.4] days). Boarding was prevalent among individuals with primary diagnoses of suicide-related behaviors and depressive disorders.

**Table.** Characteristics of Medicaid-Enrolled Youths With ED Visits for Mental Health Conditions, 2022

Characteristic	ED visits		
	Overall (N = 255 139)	With no boarding (n = 224 780)	With boarding (n = 30 359)
Patient age, mean (SD), y	14.6 (2.6)	14.5 (2.6)	14.8 (2.4)
Patient sex, No. (%)			
Male	91 013 (35.7)	80 247 (35.7)	10 766 (35.5)
Female	164 126 (64.3)	144 533 (64.3)	19 593 (64.5)
Boarding length of stay, mean (SD), d	2.1 (3.5)	1.8 (3.6)^a^	4.5 (1.4)
CCSR category prevalence, No. (%)^b^			
Suicidal ideation, attempt, or intentional self-harm	116 419 (45.6)	102 946 (45.8)	13 473 (44.4)
Anxiety and fear-related disorders	39 637 (15.5)	38 718 (17.2)	919 (3.0)
Depressive disorders	28 062 (11.0)	21 694 (9.7)	6368 (21.0)
Trauma- and stressor-related disorders	22 193 (8.7)	20 373 (9.1)	1820 (6.0)
Disruptive, impulse-control, and conduct disorders	19 617 (7.7)	17 459 (7.8)	2158 (7.1)
Other specified and unspecified mood disorders	14 720 (5.8)	11 536 (5.1)	3184 (10.5)
Schizophrenia spectrum and other psychotic disorders	6824 (2.7)	5708 (2.5)	1116 (3.7)
Bipolar and related disorders	5615 (2.2)	4554 (2.0)	1061 (3.5)
Eating disorders	824 (0.3)	703 (0.3)	121 (0.4)
Personality disorders	755 (0.3)	661 (0.3)	94 (0.3)
Obsessive-compulsive and related disorders	473 (0.2)	428 (0.2)	45 (0.1)

Abbreviations: CCSR, Clinical Classifications Software Refined; ED, emergency department.

^a^ Boarding length of stay represents the mean (SD) number of days in the ED, spanning 0 to 1 midnights. Stays spanning 0 midnights are defined as having a length of stay of 1; stays spanning 1 midnight are defined as having a length of stay of 2 days.

^b^ The CCSR groups *International Classification of Diseases, Tenth Revision, Clinical Modification* diagnosis codes into clinically meaningful categories.

Boarding rates varied substantially across states, with those in the bottom quintile having rates below 7.6% and those in the highest quintile above 15.4% (Figure). Arkansas had the lowest rate (2.7%) of mental health ED visits resulting in a boarding event, whereas Iowa had the highest (27.3%). In Montana, North Carolina, Maine, Florida, and Iowa, boarding occurred in more than 1 in 5 such visits (21.8%-27.3%).

**Figure.**
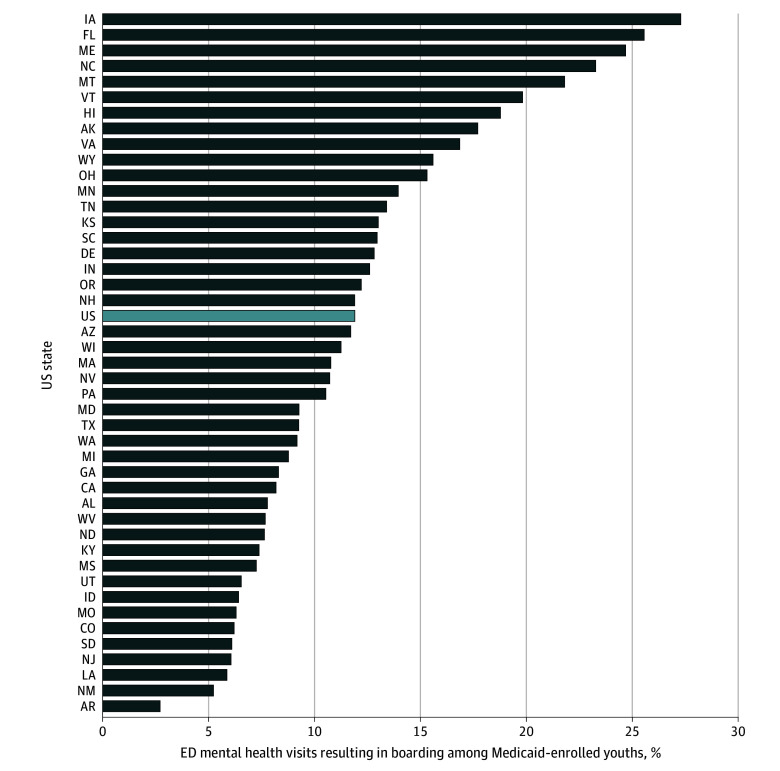
Share of Emergency Department (ED) Mental Health Visits Resulting in Boarding Among Medicaid-Enrolled Youths, 2022

## Discussion

These findings suggest that ED boarding is a considerable issue for Medicaid-enrolled youths, with more than 1 in 10 mental health–related ED visits lasting more than 2 days. In 5 states, boarding occurred in more than 1 in 5 such visits.

Boarding poses a substantial emotional toll on patients, families, and staff; it also may result in challenges related to patient and staff safety and restraint use, and it suggests an inability to find timely and appropriate care for youths in crisis. The high levels of boarding observed in our study and the substantial state-level variation should warrant concern, particularly given evidence that boarding can lead to care delays, clinical deterioration, and increased costs^5^—outcomes that may disproportionately affect low-income children who face systemic barriers to timely and coordinated behavioral health care.

A variety of factors at the individual, state, and Medicaid program levels may contribute to variations in boarding rates. These factors include the prevalence and severity of mental health conditions among youths, the generosity of Medicaid coverage, and psychiatric bed capacity.^6^

Key limitations of this cohort study are the potential for data quality or coding differences across states. We excluded boarding events lasting more than 6 midnights to minimize the risk of coding errors, potentially leading to a more conservative estimate of both the frequency and duration of boarding events. The substantial state-level differences we observed suggest that state-level policies—including an assessment of the continuum of care that includes inpatient and residential beds, subacute beds, non-ED crisis support, and accessible outpatient care—could play a key role in reducing boarding and its impact on youths and their families.

## Notes

Supplement.
Data Sharing Statement
