# Supplementary material for: Variations in Psychiatric Emergency Department Boarding for Medicaid-Enrolled Youths
Source: JAMA Health Forum. 2025 Aug 15;6(8):e253177. doi: 10.1001/jamahealthforum.2025.3177 (PMC12357182; doi:10.1001/jamahealthforum.2025.3177)
Supplement: Supplement. — Data Sharing Statement [file jamahealthforum-e253177-s001.pdf]

Data Sharing Statement

McConnell. Variations in Psychiatric Emergency Department Boarding for Medicaid-Enrolled Youths. *JAMA Health Forum*. Published August 15, 2025.
doi:10.1001/jamahealthforum.2025.3177

Data
Data available: No

Additional Information
Explanation for why data not available: Data are obtained through a third-party vendor (ResDAC) and data use agreements prohibit us from sharing these data.
